# Direct bandgap materials based on the thin films of Se_*x*_Te_100 − *x*_ nanoparticles

**DOI:** 10.1186/1556-276X-7-509

**Published:** 2012-09-15

**Authors:** Numan Salah, Sami S Habib, Zishan H Khan

**Affiliations:** 1 Center of Nanotechnology, King Abdulaziz University, Jeddah, 21589, Saudi Arabia; 2 Department of Applied Sciences, Faculty of Engineering and Technology, Jamia Millia Islamia (Central University), New Delhi, 110025, India

**Keywords:** Se_*x*_Te_100 − *x*_, Nanoparticles, Direct energy bandgap, Photoluminescence, Raman spectroscopy

## Abstract

In this study, we fabricated thin films of Se_*x*_Te_100 − *x*_ (*x* = 0, 3, 6, 9, 12, and 24) nanoparticles using thermal evaporation technique. The results obtained by X-ray diffraction show that the as-synthesized nanoparticles have polycrystalline structure, but their crystallinity decreases by increasing the concentration of Se. They were found to have direct bandgap (*E*_g_), whose value increases by increasing the Se content. These results are completely different than those obtained in the films of Se_*x*_Te_100 − *x*_ microstructure counterparts. Photoluminescence and Raman spectra for these films were also demonstrated. The remarkable results obtained in these nanoparticles specially their controlled direct bandgap might be useful for the development of optical disks and other semiconductor devices.

## Background

In the last two decades, much research work is focused on the synthesis and characterization of semiconducting nanomaterials [1]. Among these nanostructures, nanochalcogenides are important materials for various applications such as nanoelectronic devices, nanomemory devices, optical memory devices, etc. Recently, we have produced different nanochalcogenides and studied their structural, optical, and electrical properties [2-7]. However, these studies are still at the early stage and need to be further extended to cover more chalcogenide, that is, due to the primary remarkable results obtained in their nanostructure forms.

Several studies were focused on fabricating different nanostructures of different amorphous alloys/crystalline materials and studying their properties. For example, Tripathi et al. [8] have studied the optical properties of Se_100 − *x*_Te_*x*_ (*x* = 4, 8, and 16) nanostructured thin films grown by thermal evaporation. Chawla et al. [9] have synthesized Zn_1 − *x*_Cd_*x*_S:Cu nanoparticles and tuned the bandgap by increasing the Cd content. El-Nahass et al. [10] have studied the influence of heat treatment and gamma ray irradiation on the structural and optical characterizations of nanocrystalline cobalt phathalocyanine thin films. Gracin et al. [11] have analyzed the amorphous-nanocrystalline multilayer structures by optical, photodeflection, and photocurrent spectroscopies.

The studies on the preparation and characterization of nanocrystalline thin films are also available in the literature by various workers [12-14]. Keeping in view the scope of nanostructure materials, we have communicated several reports on synthesis and characterization of nanochalcogenides in both the amorphous and crystalline forms by different techniques [2-7]. Therefore, the preparation and characterization of nanochalcogenide materials are extremely important for application in optical devices. This work reports on thin films of Se_*x*_Te_100 − *x*_ (*x* = 0, 3, 6, 9, 12, and 24) nanoparticles synthesized using thermal evaporation method. The as-synthesized nanoparticle films were characterized by X-ray diffraction (XRD), scanning electron microscopy (SEM), energy dispersive spectroscopy (EDS), absorption spectrum, photoluminescence (PL), and Raman spectroscopy.

## Methods

Glassy alloy of Se_*x*_Te_100 − *x*_ (*x* = 0, 3, 6, 9, 12, and 24) in powder form have been prepared by the method adopted from Khan et al. [7]. In this method, high purity (99.999%) materials are weighed in appropriate proportions according to their atomic percentages (at.%) and sealed into quartz ampoules under vacuum of about 10^−5^ Torr. The sealed ampoules are then kept in a muffle furnace, where the temperature is raised up to 950 K at the rate of 3 K/min. Once the desired temperature of 950 K is reached, the sealed ampoules are kept at this temperature for 14 h with rocking. Through the heating process, ampoules are rotated in clockwise and anticlockwise directions with the help of the motor to ensure homogeneity of the composition within the samples. Once this process is over, the melt is rapidly quenched in ice water to make it amorphous.

Thin films of Se_*x*_Te_100 − *x*_ (*x* = 0, 3, 6, 9, 12, and 24) nanoparticles were fabricated using physical vapor condensation method. Initially, a small quantity of glassy alloy of Se_*x*_Te_100 − *x*_ in powder form is kept in a molybdenum boat, and the chamber is evacuated to a vacuum of the order of 10^−6^ Torr. After reaching this vacuum level, the argon gas is purged inside the chamber. The pressure of the gas was kept constant at 5 Torr. The glassy alloy is then evaporated in the presence of the ambient argon gas atmosphere in the chamber to get the nanostructures. The substrate is cooled with liquid nitrogen, and this evaporated material is deposited on a glass substrate pasted on this cooled substrate. The thicknesses of the films were measured using a quartz crystal monitor Edward model FTM 7 (Edwards BOC, England, UK). The thickness is fixed at 30 nm for all the films. This value was confirmed by the surface profiler AS 500 Tencor AlphaStep (Brumley South, Inc. Mooresville, NC, USA).

The as-synthesized samples were characterized by X-ray diffraction, using an Ultima-IV (Rigaku Corporation, Tokyo, Japan) diffractometer with Cu Kα radiation, while the morphology of these nanostructures is studied by SEM using Quanta, FEI (Eindhoven, The Netherlands). The chemical compositions of the deposited films were measured by the EDS technique using EDAX, Ametec. The optical absorption of thin films of Se_*x*_Te_100 − *x*_ (*x* = 3, 6, 9, and 12) have been measured by a UV-visible computerized spectrophotometer (model ‘UV-1650PC,’ Shimadzu Corporation, Tokyo, Japan) in the wavelength region of 400 to 1,100 nm. Here, we have kept the samples (films) and reference (glass substrate) in the chamber to neutralize the absorption of glass. The absorption has been measured in terms of optical density. The optical absorption is measured as a function of incidence photon energy. PL emission spectra for the thin films of Se_*x*_Te_100 − *x*_ were recorded at room temperature at excitation wavelength of 325 nm using a fluorescence spectrofluorophotometer, model RF-5301 PC, Shimadzu, Japan, while Raman spectra were measured using DXR Raman microscope, Thermo Scientific Inc. (Waltham, MA, USA) using the 532-nm laser as excitation source at 6-mW power.

## Results and discussion

**Figure 1:**
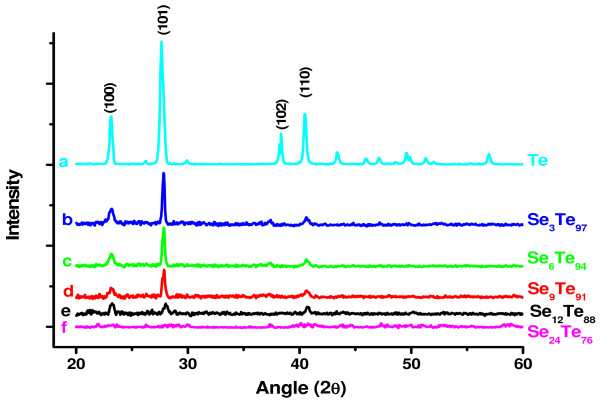
**XRD pattern of the as-synthesized thin film of Se**_***x***_**Te**_**100 −*x***_**nanoparticles.**

XRD patterns of the as-synthesized thin films of Se_*x*_Te_100 − *x*_ (*x* = 0, 3, 6, 9, 12, and 24) are presented in Figure 1 (curves a, b, c, d, e, and f). The figure shows few structural diffraction peaks, which might be due to the presence of a mixture of amorphous and crystalline phases. The crystalline phase is found to decrease by increasing the concentration of Se. At the highest concentration of Se (i.e., *x* = 24), the material is completely amorphous (Figure 1f), while that of *x* = 0 (Figure 1a), it has almost crystalline structure. It has been reported by several authors that the XRD pattern of pure Te shows high degree of crystallinity [15,16], while selenium (Se) easily forms amorphous phase [17,18]. This is in agreement with the present result where pure Te shows crystalline phase, and its alloying with Se reduces the crystallinity of Se_*x*_Te_100 − *x*_ system.

**Figure 2:**
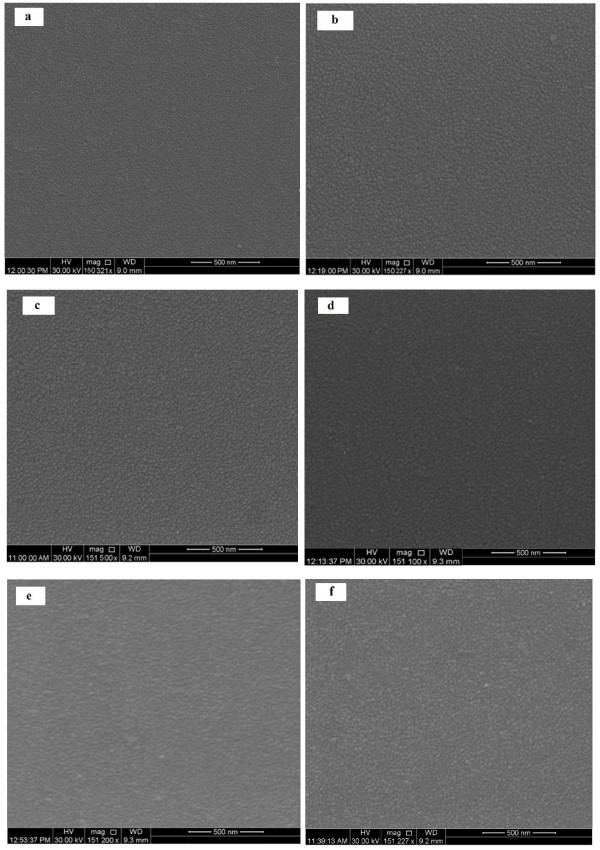
**SEM images for the thin films of Se**_***x***_**Te**_**100 −*x***_**nanoparticles.** (**a**) Te, (**b**) Se_3_Te_97_, (**c**) Se_6_Te_94_, (**d**) Se_9_Te_91_, (**e**) Se_12_Te_88_, and (**f**) Se_24_Te_76_.

SEM images for the as-synthesized thin films of Se_*x*_Te_100 − *x*_ are presented in Figure 2a,b,c,d,e,f for Te, Se_3_Te_97_, Se_6_Te_94_, Se_9_Te_91_, Se_12_Te_88_, and Se_24_Te_76_, respectively. Small nanoparticles can be seen in these films with particle size in the range of 15 to 20 nm. The morphology of these nanoparticles is almost similar in all the films. The particle size in pure Te film (Figure 2a) is smaller than those grown in Se-Te films.

**Figure 3:**
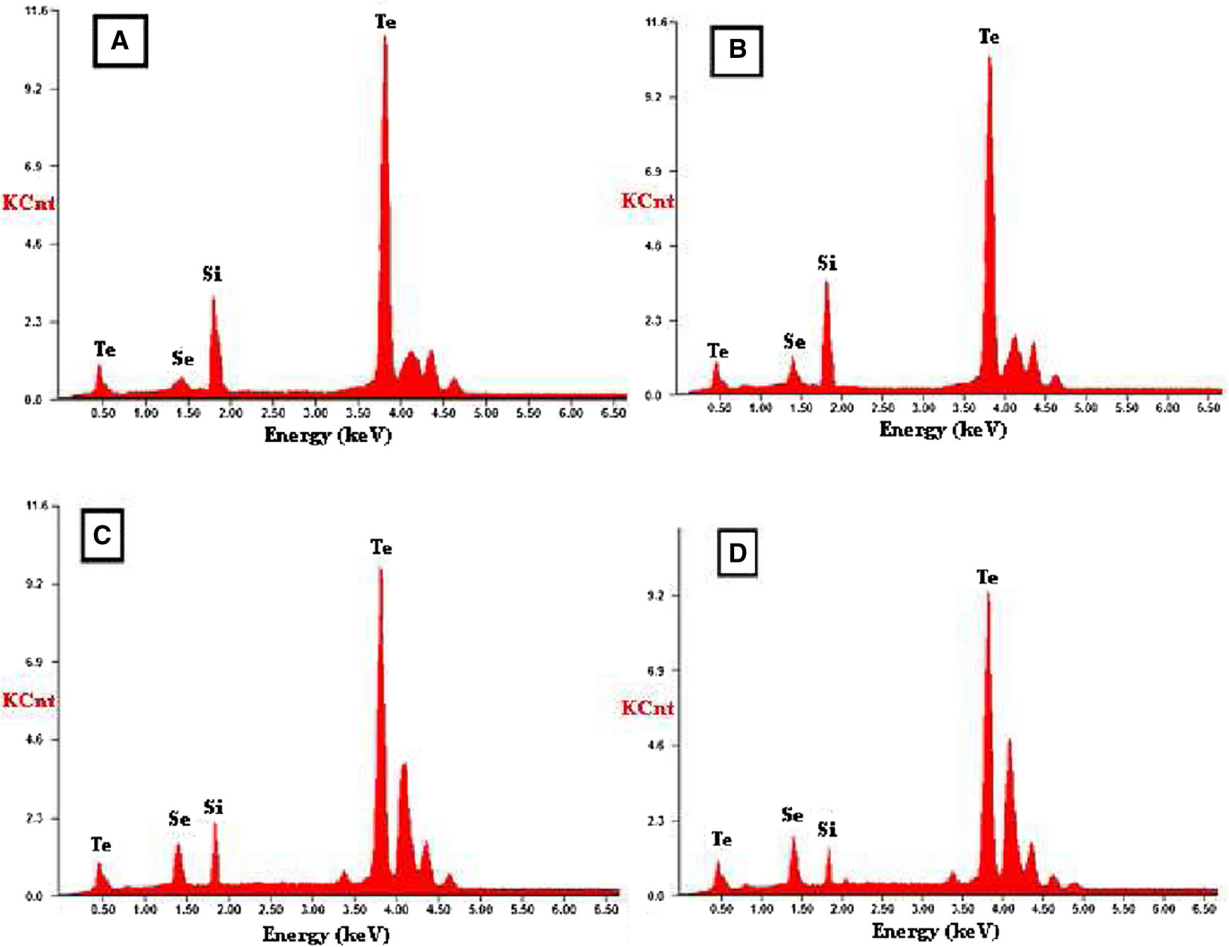
**Typical EDS results for the thin films of nanoparticles.** (**A**) Se_3_Te_97_, (**B**) Se_6_Te_94_, (**C**) Se_9_Te_91_, and (**D**) Se_12_Te_88_ nanoparticles.

The chemical compositions of these films were measured using EDS. Typical results obtained from some of these films are presented in Figure 3A,B,C,D. The EDS results show that the deposited films have compositions close to those of the bulk materials. This figure shows clearly the decrease in Te and increase in Se concentrations.

**Figure 4:**
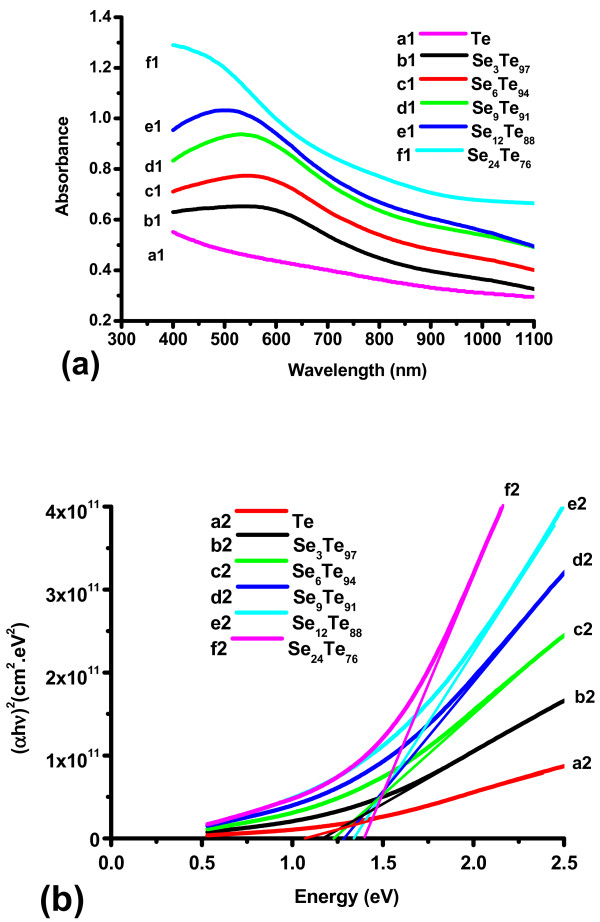
**UV-visible absorption spectra (a) and (*α*hv)**^**2**^**against photon energy (*hν)*(b).** For the thin films of Se_*x*_Te_100 − *x*_ nanoparticles.

Figure 4a (curves a1, b1, c1, d1, e1, and f1) shows the UV-visible absorption spectra of the as-synthesized thin films of Se_*x*_Te_100 −__*x*_ nanoparticles. The curves of Se-Te films show maximum absorption bands at around 520 nm, while that of pure Te has lower absorption around this value. The absorption increases by increasing the concentration of Se. This might be due to the decrease in the crystallinity of the deposited material as could be seen from the XRD results (Figure 1). The value of the absorption coefficient (*α*) has been calculated using the following relation:

(1) α=OD/t

where OD is the optical density measured at a given layer thickness *t*. This *α* is used to obtain the energy bandgap (*E*_g_) values. The variation of *α* with incident photon energy (hν*)* in the present thin films of Se_*x*_Te_100 −__*x*_ nanoparticles was found to obey the rule of direct transition, and the relation between the optical gap, optical absorption coefficient, and the energy hν of the incident photon is given by [7]:

(2) $${\left(\alpha h\nu \right)}^{2}\propto \left(h\nu -E_{g}\right)$$

Figure 4b shows the variation of (*α*hν)^2^ with photon energy (hν) for the thin films of Se_*x*_Te_100 − *x*_ nanoparticles (curves a2, b2, c2, d2, e2, and df). The value of direct optical bandgap (*E*_g_) is calculated by taking the intercept on the *x*-axis. The calculated values of *E*_g_ for the thin films of Te, Se_3_Te_97_, Se_6_Te_94_, Se_9_Te_91_, Se_12_Te_88_, and Se_24_Te_76_ are 1.08, 1.17, 1.22, 1.28, 1.34, and 1.39 eV, respectively. It is clear that there is a significant increase in the value of the optical bandgap by increasing the Se concentration in this system. These values are comparable with those of Se_*x*_Te_100 − *x*_ microsize alloys presented by Khan et al. [7]. These films of Se_*x*_Te_100 − *x*_ microsize particles were reported to have indirect bandgaps with values decreasing by increasing the concentration of Se [7]. They have attributed this decrease to the increase of the localized states in the amorphous systems due to Se addition.

**Figure 5:**
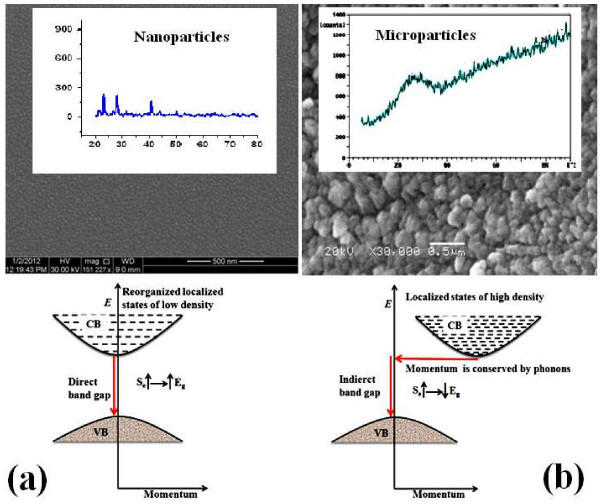
**Schematic band structure of Se**_***x***_**Te**_**100 −*x***_**(a) nano- and (b) microsize particles.** SEM and XRD results of the microsize particles are obtained from [9].

In the present nanoscale materials, if we compare the results presented by Khan et al. [7] with our findings, it is clear that their microsize materials are completely of amorphous nature, which is not the case in the present nanomaterials. The thin films of Se_*x*_Te_100 − *x*_ nanoparticles have a considerable amount of crystalline phase (Figure 1) that might have lesser concentration of localized states (lower density of localized states than those at the microsize). These localized states induced in the band structure of Se_*x*_Te_100 − *x*_ nanoparticles might have reorganized their positions during the nanoparticle growth, result in a formation of direct band gap. These reorganized localized states of lower density might also be responsible for the increase in values of the bandgap by adding Se to the host. It means that at the nanoscale level, particularly at sizes closer to quantum dots, the energy levels might arrange themselves to have direct bandgap. Further addition of dopants (defects/localized states) to the host could increase the value of this bandgap. These behaviors are completely different than those of the microsize particles. These illustrations are presented in Figure 5. This figure shows clearly the direct and indirect bandgaps formed in the nano- and microsize particles of the thin films of Se_*x*_Te_100 − *x*_ (Figure 5a,b, respectively). The figure also shows the conduction bands in both cases, which have different densities of localized states due to the crystallinity present in these structures. Typical SEM images and XRD results of these nano- and microsize particles are also shown in Figure 5 (SEM image and XRD result of the microsize particles are obtained from [7]).

Tripathi et al. [8] synthesized nanostructures of selenium-rich samples (Se_100 − *x*_Te_*x*_, *x* = 4, 8,16) in the presence of oxygen and argon, but their results show indirect bandgap in their nanoparticle films (their particle size is in the range of 40 to 100 nm). This might be due to the size of their nanoparticles, which is much bigger than that of the quantum dots. The other possibility is that tellurium atom (0.140 nm) as dopants, which is bigger than Se atom (0.115 nm), could not induce defects/localized states that have the ability to arrange themselves to provide direct bandgap. In the present case, it is the remarkable results for tellurium-rich compounds in nanostructure form to have direct bandgaps. This might be attributed to the reorganization and modifications induced in positions of the localized states of the nanostructure materials as explained above.

Since the optical absorption depends on short-range order in the amorphous states and defects associated with it, the change in optical bandgap of the films of microsize particles of Se_*x*_Te_100 − *x*_ was explained by Khan et al. [7] on the basis of ‘density of state model’ proposed by Mott and Davis [19]. According to this model, the width of the localized states near the mobility edges depends on the degree of disorder and defects present in the amorphous structure. In particular, it is known that unsaturated bonds together with some saturated bonds are produced as the result of an insufficient number of atoms deposited in the amorphous film [20]. The unsaturated bonds are responsible for the formation of some of the defects in the films, producing localized/defect states in the amorphous solids. However, this model [19] has not taken into consideration the effect of particle size on these localized states. In addition to this model, it is expected that at the nanoscale level these localized/defect states might have reorganized their positions in the band structure, resulting in the formation of direct bandgap (Figure 5). Crystal field effect might be another factor affecting the localized states formed in the nanoparticles, which might be smaller than that of the microsize particles, resulting in the widening of the bandgap. This increase in optical bandgap may also be due to the shift in Fermi level whose position is determined by the distribution of electrons over the localized states [21].

**Figure 6:**
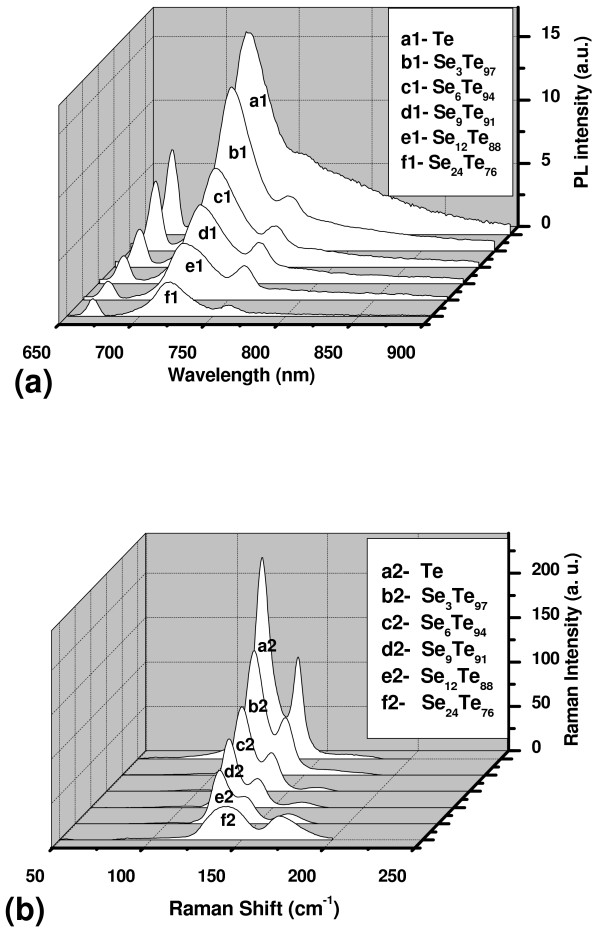
**Photoluminescence emission spectra (a) and (b) Raman spectra.** Of the thin films of Se_*x*_Te_100 − *x*_ nanoparticles.

PL emission spectra for the as-synthesized thin films of Se_*x*_Te_100 − *x*_ nanoparticles are presented in Figure 6a (curves a1, b1, c1, d1, e1, and f1). These films were excited by 325 nm. Three emission bands are observed at around 666, 718, and 760 nm in these films. The second band at 718 nm is over two times stronger than those at 666 and 760 nm. These bands are observed in the PL spectra of all the films deposited at different values of *x*. The only change that could be observed by increasing the concentration of Se is a reduction in the intensities of these bands. The intensities of these bands were significantly decreased by increasing the dopant concentration. The PL emission spectrum is similar to those observed in single crystals of GaTe reported by several authors [22-24]. It is therefore possible that the origin of these bands mainly the first and second bands is similar to those observed in the single crystal of GaTe, which are the recombination of direct free excitons (radiative annihilation of free excitons) and the impurity levels (such as residual impurity, defects, or defect complexes) localized in the forbidden energy gap. The second band at 718 nm might be attributed to the recombination of direct free excitons [22,23], while that at 666 nm might be ascribed to the impurity levels localized in the forbidden energy gap. The third band at 760 nm may be due to the recombination of direct free excitons at the surface of the grown nanoparticles. The intensities of these bands might have a direct link to the degree of crystallinity. In the present alloy, it is possible that the recombination of some of the free excitons becomes non-radiative by going from crystalline to amorphous phase. This might be the reason for the PL quenching. Moreover, it is quite possible that there might be a recombination of free excitons located at the adjacent nanoparticles. Electrons in one nanoparticle might have recombined with the holes of the next nanoparticle. In this case, the flux or the density of the emitted photons might be lower than those in the case of single crystals. This might also result in PL quenching.

Raman spectra of the thin films of Se_*x*_Te_100 − *x*_ nanoparticles are presented in Figure 6b (curves a2, b2, c2, d2, e2, and d2). The most noticeable features in the Raman spectra of these films are the three bands observed at around 123, 143, and 169 cm^−1^. The first band at 123 cm^−1^ is the most prominent one. Intensities of the first two bands are observed to decrease by increasing the concentration of Se, while it is vice versa for the band observed at 169 cm^−1^. The band at 123 cm^−1^ might be assigned to the A_1_ mode corresponding to symmetric stretching of a triangle of three Te atoms [25,26], while that at 143 cm^−1^ might be ascribed to the amorphous Te-Te stretching mode [27,28]. The later one has also been assigned to t-Se crystals [29]. The band at around 169 cm^−1^ might be assigned to the Se-Se bonds [30]. The intensity of this band is significantly increased by increasing the concentration of Se. These results show that the progressive introduction of selenium to the structure of tellurium in the alloys of S_*x*_Te_100 − *x*_ induces the breaking of Te-Te bonds (especially those of the crystalline phase). These results are consistent with those of the XRD (Figure 1). The most intense band at 123 cm^−1^ is significantly reduced by adding Se as a result of decreasing the crystallinity of the system.

From the application point of view, the obtained results in the present tellurium rich nanomaterials (Se_*x*_Te_100 − *x*_, *x* = 0, 3, 6, 9, 12, and 24) are remarkable. The results show the controlled direct bandgap, which might be useful for different applications. This direct bandgap gives more favorable optoelectronic properties than the indirect bandgap [31]. Direct bandgap means that electrons at the minimum of the conduction band have the same momentum as electrons at the maximum of the valence band, and for an indirect bandgap, the electrons do not have the same momentum. The recombination of an electron near the bottom of the conduction band with a hole near the top of the valence band requires the exchange of energy and momentum. For indirect bandgap recombination, the energy may be carried off by a photon, but one or more phonons are required to conserve momentum (Figure 5). This multiparticle interaction is improbable, and the recombination efficiency in the indirect bandgap material is lower than that in the case of direct bandgap material. The majority part of semiconductors is indirect bandgap material; compared with them, direct bandgap materials are preferred for several applications such as laser diodes. Direct bandgap structures maximize the tendency of electrons and holes to recombine by stimulated emission, thus increasing the laser efficiency. Moreover, the material with controlled bandgap (by increasing the concentration of Se in the present nanomaterial) expected to have better properties. They can be alloyed to ternary and quaternary compositions, with adjustable bandgap width. Here in the present nanomaterials, i.e., Se_*x*_Te_100 − *x*_, there are no changes in positions of the emitted wavelengths (Figure 6a) due to changing the bandgap values. The only change that could be observed is the intensity of these emissions. Thus, controlling the intensity of these emissions might be useful in optoelectronic devices. Furthermore, providing materials with wide bandgaps allows operation of power devices at higher temperatures and gives lower thermal noise to low-power devices at room temperature [32].

## Conclusions

Thin films of Se_*x*_Te_100 − *x*_ (*x* = 0, 3, 6, 9, 12, and 24) nanoparticles with particle size in the range of 15 to 20 nm have been synthesized using thermal evaporation technique. The as-grown nanoparticles have polycrystalline structure, but their crystallinity decreases by increasing the concentration of Se. These nanomaterials are found to have direct bandgap, which differ from their microstructure counterparts. Moreover, this bandgap could be tuned by changing the concentration of Se. PL emission spectra for these films showed three bands at 666, 718, and 760 nm, while Raman spectra display three bands at 123, 143, and 169 cm^−1^. The intensities of PL and Raman bands were decreased by increasing the concentration of Se except that of the last band of Raman wherein it is increased. These results might be useful for the development of optical disks and other semiconducting devices based on these controlled direct bandgap nanomaterials.

## Competing interests

The authors declare that they have no competing interests.

## Authors' contributions

All authors equally contributed in writing the manuscript and in performing the experiments. All authors read and approved the final manuscript.

## References

[B1] Kuo K Hsu S Huang P Chuang W Liu C Tsung Lee PO Optical properties and sub-bandgap formation of nano-crystalline Si quantum dots embedded ZnO thin film Opt Express 2012 20 10470 10.1364/OE.20.010470 22565671

[B2] Khan ZH Khan SA Salah N Habib S El Hamidy SMA Al-Ghamdi AA Effect of composition on electrical and optical properties of thin films of amorphous Ga_x_Se_100-x_ nanorods Nanoscale Res Lett 2010 5 1512 10.1007/s11671-010-9671-5 20730131 PMC2920423

[B3] Khan ZH Khan SA Habib S Al-Ghamdi AA Salah N Morphology and optical properties of thin films of Ga_x_Se_100 - x_ nanoparticles Nanosci Nanotechnol Lett 2011 3 319 10.1166/nnl.2011.1188

[B4] Khan ZH Khan SA Salah N Habib SS Al-Ghamdi AA Electrical transport properties of thin film of a-Se_87_Te_13_ nanorods J Exp Nanosci 2011 6 337 10.1080/17458080.2010.497946

[B5] Khan ZH Khan SA Salah N Al-Ghamdi AA Habib SS Electrical properties of thin films of a-Ga_x_Te_100− x_ composed of nanoparticles Phil Mag Lett 2011 91 207 10.1080/09500839.2010.547227

[B6] Salah N Habib SS Khan ZH Alarfaj E Khan SA Synthesis and characterization of Se_35_Te_65 –*x*_Ge_*x*_ nanoparticle films and their optical properties J Nanomater 2012 201 2 393084

[B7] Khan ZH Salah N Habib SS Al-Ghamdi AA Khan SA Electrical and optical properties of a-Se_x_Te_100–x_ thin films Opt Laser Technol 2012 4 6

[B8] Tripathi K Bahishti AA Khan MAM Husain M Zulfequar M Optical properties of selenium–tellurium nanostructured thin film grown by thermal evaporation Physica B 2009 404 2134 10.1016/j.physb.2009.03.049

[B9] Chawla AK Singhal S Nagar S Gupta HO Chandra R Study of composition dependent structural, optical, and magnetic properties of Cu-doped Zn_1−*x*_Cd_*x*_S nanoparticles J Appl Phys 2010 108 123519 10.1063/1.3524516

[B10] El-Nahass MM Farag AAM Atta AA Influence of heat treatment and gamma-rays irradiation on the structural and optical characterizations of nano-crystalline cobalt phthalocyanine thin films Synthetic Met 2009 159 589 10.1016/j.synthmet.2008.11.029

[B11] Gracin D Sancho-Paramon J Juraić K Gajović A Čeh M Analysis of amorphous-nano-crystalline multilayer structures by optical, photo-deflection and photo-current Micron 2009 40 56 10.1016/j.micron.2008.03.011 18502137

[B12] Obey K Abdul Khadar M Evolution of nanostructure, defect-free photoluminescence and enhanced photoconductivity of oxidized Zn films J Appl Phys 2011 109 124315 10.1063/1.3592650

[B13] Kuo D-H Chang B-J Growth behaviors of ZnO nanorods grown with the Sn-based bilayer catalyst-covered substrates J. Nanomater 2011 201 1 603098

[B14] Amin G Asif MH Zainelabdin A Zaman S Nur O Willander M Influence of pH, precursor concentration, growth time, and temperature on the morphology of ZnO nanostructures grown by the hydrothermal method J Nanomater 2011 201 1 269692

[B15] Haoyong Y Zhude X Jingyi B Huahui B Yifan Z Ethylenediamine assisted growth of single crystal tellurium channels Materials Letters 2005 59 3779 10.1016/j.matlet.2005.07.015

[B16] Brian M Younan X One-dimensional nanostructures of trigonal tellurium with various morphologies can be synthesized using a solution-phase approach J. Mater. Chem 1875 2002 12

[B17] Soliman AA El-Nahass MM Glass transition behavior of binary Ga_*x*_Se_100−*x*_ (0 ≤ *x* ≤ 10) glass systems Physica B: Condensed Matter 2008 403 3331 10.1016/j.physb.2008.04.031

[B18] Abhay Kumar S Neearj M Kedar S Optical and FTIR properties of Se_93−*X*_Zn_2_Te_5_In_*X*_ chalcogenide glasses Physica B: Condensed Matter 2009 404 3470 10.1016/j.physb.2009.05.045

[B19] Mott NF Davis EA Electronics Processes in Non-Crystalline Materials 1979 Oxford: Clarendon

[B20] Theye ML Proceedings of the 5th International Conference on Amorphous and Liquid Semiconductors 1973 1 Germany: Garmisch-Partenkirchen London: Taylor and Francis

[B21] Nang TT Okuda M Matsushita T Yokota S Suzuki A Electrical and optical properties of Ge_*x*_Se_1-*x*_ amorphous thin films Jpn J Appl Phys 1976 15 849 10.1143/JJAP.15.849

[B22] Taylor RA Ryan JF Time-resolved exciton photoluminescence in GaSe and GaTe J Phys C 1987 20 6175 10.1088/0022-3719/20/36/018

[B23] Güder HS Abay B Efeoğlu H Yoğurtu YK Photoluminescence characterization of GaTe single crystals J Lumin 2001 93 243 10.1016/S0022-2313(01)00192-2

[B24] Shigetomi S Ikari T Nishimura H Temperature dependence of photoluminescence of layer semiconductor p-GaTe J Lumin 1998 78 117 10.1016/S0022-2313(97)00305-0

[B25] Sridharan M Narayandass SK Mangalaraj D Lee HC Sridharan M Narayandass SK Mangalaraj D Lee HC Raman scattering studies on B^+^ implanted Cd_0.96_Zn_0.04_Te thin films Vacuum 2003 68 119

[B26] Sen S Gjersing EL Aitken BG Physical properties of Ge_x_As_2x_Te_100−3x_ glasses and Raman spectroscopic analysis of their short-range J Non-Cryst Solids 2010 356 2083 10.1016/j.jnoncrysol.2010.08.013

[B27] Tominaga J Atoda N Study of the crystallization of GeSbTe films by Raman spectroscopy Jpn J Appl Phys 1999 38 L322 10.1143/JJAP.38.L322

[B28] Wu Y Liu K Li D Guo Y Pan S In situ AFM and Raman spectroscopy study of the crystallization behavior of Ge_2_Sb_2_Te_5_ films at different temperature Appl Surf Sci 2011 258 1619 10.1016/j.apsusc.2011.10.021

[B29] Holubová J Černošek Z Černošková E The selenium based chalcogenide glasses with low content of As and Sb: DSC, StepScan DSC and Raman spectroscopy study J Non-Cryst Solids 2050 2009 355

[B30] Pan RK Tao HZ Zang HC Zhao XJ Zhang TJ Annealing effects on the structure and optical properties of GeSe_2_ and GeSe_4_ films prepared by PLD J Alloys Compd 2009 484 645 10.1016/j.jallcom.2009.05.011

[B31] Teich MC Saleh BEA Fundamentals of Photonics 1991 1 New York: Wiley

[B32] Neudeck Philip G Okojie Robert S Chen L-Y High-temperature electronics—a role for wide bandgap semiconductors? Proceedings of the IEEE 2002 90 1065 10.1109/JPROC.2002.1021571

